# Identification of Nucleophilic Probes for Protease-Mediated Transpeptidation

**DOI:** 10.3390/molecules23092109

**Published:** 2018-08-22

**Authors:** Ga-eul Eom, Seokhee Kim

**Affiliations:** Department of Chemistry, Seoul National University, 1 Gwanak-ro, Gwanak-gu, Seoul 08826, Korea; autumn1023@snu.ac.kr

**Keywords:** protease, transpeptidation, peptide ligation, nucleophilic probe, acyl-enzyme intermediate, DegP

## Abstract

Proteases have evolved to mediate the hydrolysis of peptide bonds but may perform transpeptidation in the presence of a proper nucleophilic molecule that can effectively compete with water to react with the acyl-enzyme intermediate. There have been several examples of protease-mediated transpeptidation, but they are generally inefficient, and little effort has been made to systematically control the transpeptidation activity of other proteases with good nucleophiles. Here, we developed an on-bead screening approach to find a probe that functions efficiently as a nucleophile in the protease-mediated transpeptidation reaction, and we identified good probes for a model protease DegP. These probes were covalently linked to the C-termini of the cleaved peptides in a mild condition and made the selective enrichment of ligated peptides possible. We suggest that good nucleophilic probes can be found for many other proteases that act via acyl-enzyme intermediates, and these probes will help characterize their substrates.

## 1. Introduction

Proteases, one of the largest groups of enzymes in all organisms, are involved in diverse cellular processes by irreversibly cleaving peptide bonds. They represent potential drug targets for many human diseases [1,2,3]. Based on which amino acid or atom is used to generate a nucleophile to attack the peptide carbonyl group, proteases are classified into six groups which have two general mechanisms of peptide hydrolysis. One is to use the hydroxyl or sulfhydryl group in a side chain as a nucleophile to make acyl-enzyme intermediates, as shown in serine, cysteine, and threonine proteases, and the other is to use an activated water molecule as a nucleophile to directly hydrolyze the peptide bonds, as presented in aspartic-, glutamic-, and metallo-proteases.

**Figure 1:**
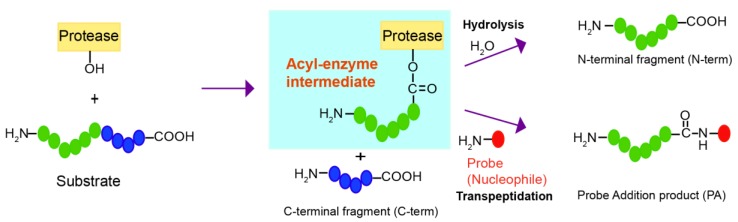
Reaction scheme of a protease that mediates either hydrolysis or transpeptidation of a peptide bond via the acyl-enzyme intermediate.

Although proteases have evolved to catalyze the efficient hydrolysis of peptide bonds, those making acyl-enzyme intermediates may also mediate transpeptidation or peptide ligation if amine nucleophiles react with the acyl-enzyme intermediate and induce aminolysis instead of hydrolysis (Figure 1). Transpeptidation has been observed under normal conditions for protein digestion with several proteases, such as trypsin, V8 protease, Glu-C, and bleomycin hydrolase [4,5,6,7,8]. By introducing several mutations, subtilisin was converted from a protease to a peptide ligase, subtiligase, which can mediate the selective biotin labeling of α-amines of peptides over ε-amines of lysines and has been applied to a proteomic approach to globally identify proteolytic cleavage sites [9,10,11]. Notable cases of transpeptidation by a protease were reported with human proteasome, called peptide splicing, in which non-contiguous peptides from the proteasomal degradation were ligated and presented as major histocompatibility complex (MHC) class I antigens on the cell surface [12,13,14,15]. However, these examples are dependent on activated substrates or nucleophiles that are generated in situ, and they are generally very inefficient processes in which hydrolysis dominates. It is also unknown whether it is possible to find a good nucleophile that will efficiently react with acyl-enzyme intermediates under mild conditions.

Here, we developed a strategy to find a good nucleophilic probe for protease-mediated transpeptidation using peptide libraries and on-bead screening. This method led to the identification of probes that are efficiently ligated to the C-termini of the peptides forming acyl-enzyme intermediates with the model DegP protease. The biotin tag in the probes allowed selective purification of the ligated peptides among various cleavage products.

## 2. Results

### 2.1. The DegP Protease Can Induce Transpeptidation

DegP, a highly-conserved protease in the bacterial periplasm that is also known as an HtrA protease, plays an important role in removing misfolded proteins [16]. DegP is a good model protease to study protease-mediated transpeptidation because, while the transpeptidation products of other proteases are normally subjected to cleavage by the same proteases in the prolonged reaction, those of DegP are not, because DegP preferentially recognizes and cleaves substrates that contain two distant degrons, the cleavage site degron, and the C-terminal hydrophobic degron [17,18,19]. The transpeptidation products of the DegP protease retain the cleavage site degron but lose the C-terminal degron.

**Figure 2:**
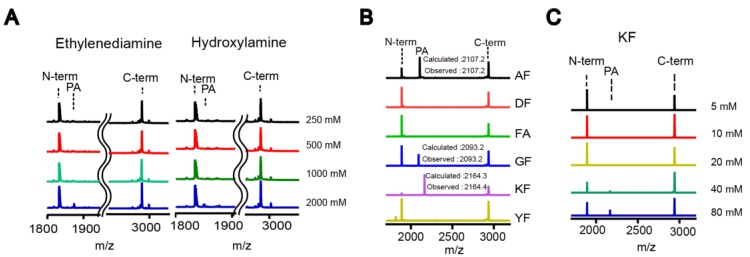
The DegP protease can mediate transpeptidation with proper nucleophiles. (**A**) A model substrate (18–58; 20 µM) was cleaved by DegP (2 µM) in the presence of ethylenediamine or hydroxylamine (0.25–2 M), and the reaction products were analyzed by MALDI-TOF-MS (N-term, N-terminal fragment; C-term, C-terminal fragment; PA, probe-addition product). Representative spectra are shown (n = 3). (**B**,**C**) The MALDI analysis of the products of the same cleavage reactions as in (**A**), but in the presence of various dipeptides (200 mM; (**B**); n = 1) or various concentrations of the KF dipeptide (5–80 mM; (**C**)). Representative spectra are shown in (**C**) (n = 3).

To test if DegP can mediate transpeptidation in the presence of a nucleophile, we initially added various concentrations of hydroxylamine (p*K*_a_ ~ 5.9) or ethylenediamine (p*K*_a_ ~ 7.5 and 10.0) in the cleavage of a model substrate (18–58) in which the peptide bond between alanine and lysine is known to be the major cleavage site, and we analyzed the reaction products with MALDI-TOF-MS (Figure S1) [19]. They showed a detectable peak for the addition product at 2 M, whereas the reactions that were devoid of the nucleophile, enzyme, or both nucleophile and enzyme did not display the peak for the addition product, indicating that the DegP protease can indeed mediate transpeptidation albeit with a very high concentration of these nucleophiles, and that the new peak did not result from the additional hydrolysis of the substrate or substrate degradation under the assay conditions (Figure 2A). To find a better nucleophile that can function at a lower concentration, we tested several dipeptides at 200 mM and found that AF, GF, and KF resulted in significant levels of the addition products (Figure 2B). Because KF showed the highest peak for the addition product compared to the N-terminal and C-terminal cleavage products, we tested lower concentrations of KF, of which 40 mM KF was sufficient to show a detectable peak for the addition product (Figure 2C). Therefore, we concluded that DegP can also mediate transpeptidation in which dipeptides function as efficient nucleophiles.

### 2.2. Screening of Dipeptide Libraries Reveals an Efficient Nucleophile for the DegP-Mediated Transpeptidation

**Figure 3:**
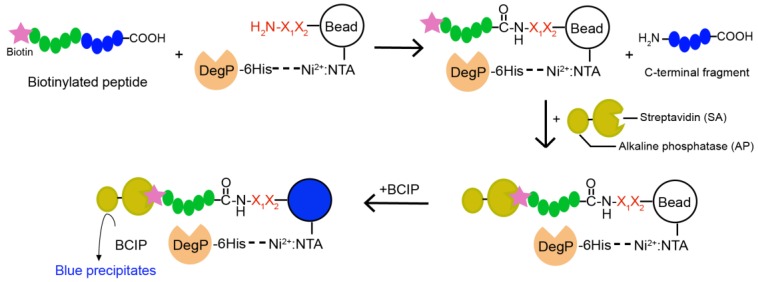
Scheme of the one-bead-one-compound (OBOC)-based approach to find a good nucleophile for the DegP-mediated transpeptidation. Beads are linked to the dipeptide library (X_1_X_2_) as well as Ni^2+^: NTA, of which the latter recruits the His-tagged DegP on beads. When the N-terminally biotinylated substrate is mixed with the above beads, beads with good nucleophiles may be covalently linked to the N-terminal fragment of the model peptide. The fusion enzyme of streptavidin and alkaline phosphatase binds to these biotinylated beads and the reaction with 5-bromo-4-chloro-3-indoyl-phosphate (BCIP) turns these beads blue.

To unbiasedly find a dipeptide nucleophile that efficiently induces transpeptidation, we designed a method using one-bead-one-compound (OBOC) peptide libraries (Figure 3) [20,21,22]. The OBOC libraries were composed of beads carrying various peptides in which the N-terminal’s two amino acids (the X_1_ and X_2_ positions from N-terminus) were randomized and followed by a linker sequence AAARM in which the three alanines, one arginine, and the C-terminal methionine were used for the longer length, solubility and better detection in MALDI, and the cyanogen bromide (CNBr)-induced cleavage of attached peptides, respectively. The split-mix synthesis of peptide libraries ensured that each bead contained only one peptide sequence that functioned as a nucleophile. To maximize the access of peptide nucleophiles to the enzyme, we induced the reaction mostly on the bead surface by attaching DegP to beads via an interaction between the His-tag and Ni^2+^-complexed nitrilotriacetic acid (Ni^2+^: NTA). When DegP-containing OBOC libraries were mixed with a model substrate carrying an N-terminal biotin, the beads with good nucleophiles were labeled with biotin by transpeptidation and detected by blue precipitates on beads which were generated by the reaction of biotin-bound streptavidin-alkaline phosphatase (SAAP) with its substrate, 5-bromo-4-chloro-3-indoyl-phosphate (BCIP).

**Figure 4:**
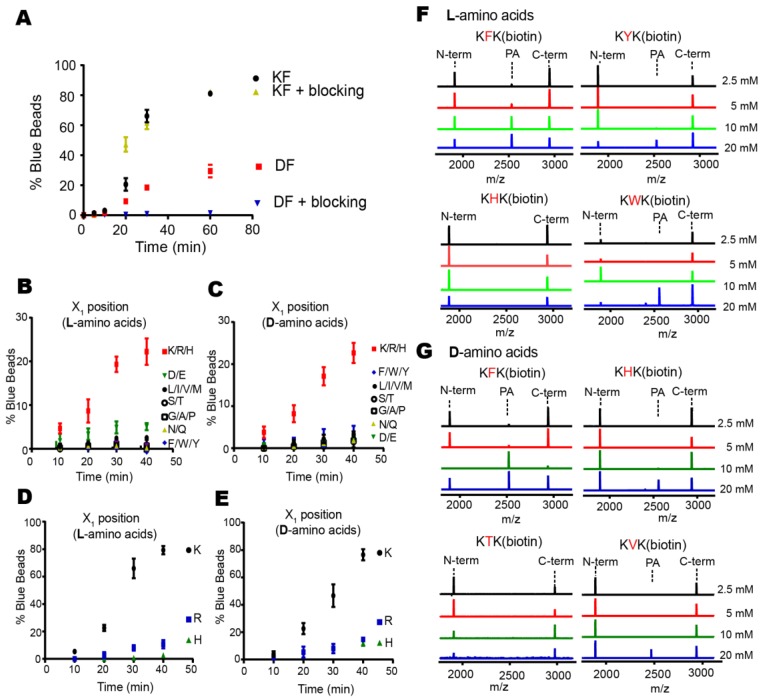
The OBOC-based approach allows identification of good nucleophiles. (**A**) Beads containing the KF dipeptide showed much higher fractions of blue beads than those with DF in the optimized on-bead reaction. The blocking of beads with bovine serum albumin (BSA) helped to reduce the background blue beads with DF. The error bars show averages ±1 SD (n = 3). (**B**,**C**) Seven groups of OBOC libraries carrying the N-terminal amino acids of similar properties were individually subjected to the on-bead screening experiments. Only the library with positive side-chains (K, R, and H) showed a significant fraction of blue beads. Peptide libraries with only l-amino acids (**B**) or d-amino acids (**C**) were used. The error bars show averages ±1 SD (n = 3). (**D**,**E**) Three OBOC libraries containing N-terminal lysine, arginine, or histidine were individually subjected to the on-bead screening experiments. Only the beads with the N-terminal lysine showed significant blue beads. The peptide libraries with only l-amino acids (**D**) or d-amino acids (**E**) were used. The error bars show averages ±1 SD (n = 3). (**F**,**G**) Various peptides bearing the identified dipeptides from the sequencing of blue beads were synthesized in the X_1_X_2_K(biotin) context and subjected to the cleavage reactions (2 µM DegP and 20 µM 18–58) in different concentrations. The reaction mixtures were analyzed by MALDI-TOF-MS (n = 1). Peptides with only l-amino acids (**F**) or d-amino acids (**G**) were used.

We initially used two dipeptides, KF and DF, as positive and negative controls for optimizing the on-bead reaction (Figure 2B or Figure 4A). As expected, more KF-beads appeared blue than DF-beads, and in particular, the blocking of beads with bovine serum albumin (BSA) reduced the background blue color only with DF-beads in the optimized on-bead reaction (Figure 4A or Figure S2). Next, we used the same reaction condition to screen OBOC libraries for transpeptidation. We prepared seven groups of libraries with l-amino acids, in which the N-terminal amino acids had similar properties, subjected them to the on-bead screening experiments, and found that only the group containing positive side chains (K, R, and H) at the N-terminus showed a significantly higher number of blue beads (Figure 4B). The experiment with only the d-amino acids also revealed the same result (Figure 4C). We further screened each library that contained N-terminal lysine, arginine, or histidine, and found that the library with N-terminal lysine showed a high number of blue beads, no matter whether l- or d-amino acids were used (Figure 4D,E). This result suggests that lysine is preferred at the N-terminal (X_1_) position for efficient transpeptidation.

To identify the preferred amino acid at the second (X_2_) position from the N-terminus, we isolated blue beads, subjected them to CNBr cleavage, and analyzed the sequence of the cleaved peptides by MALDI-TOF-MS. Out of nine peptides with l-amino acids, we obtained six KF and one of either KY, KH, or KW (Figure S3). Also, we obtained six KF, two KV, and one of either KH or KT from ten peptides with d-amino acids (Figure S3). To validate the transpeptidation by these peptides, we synthesized the X_1_X_2_K(biotin) peptides, in which the above dipeptides were attached to the *N*-ε-biotinyl-lysine, K(biotin), at C-termini for affinity purification (see below). When several concentrations of these peptides (2.5, 5, 10, and 20 mM) were tested for transpeptidation, we found that KFK(biotin) showed the most efficient addition, in which even 2.5 mM of nucleophiles showed a detectable peak for the addition product (Figure 4F,G). Collectively, we successfully applied the OBOC method to optimize nucleophiles and found that KF was the most efficient dipeptide that contained either all l-amino acids or d-amino acids.

### 2.3. KFK(Biotin) or its Close Derivative Are Good Nucleophilic Probes

**Figure 5:**
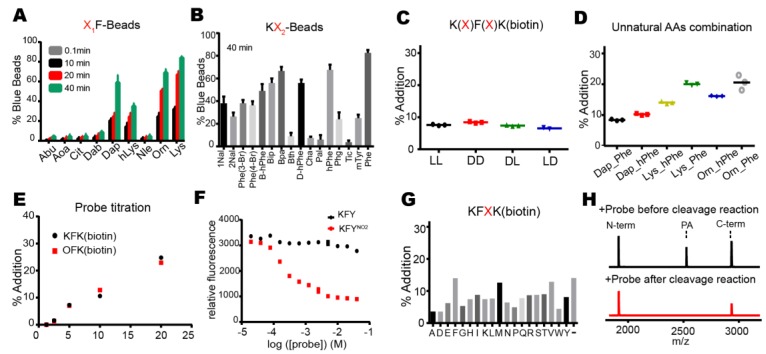
KFK(biotin) and OFK(biotin) are the best nucleophiles among those tested. (**A**) Various unnatural amino acids including the lysine mimics at the X_1_ position of the X_1_F beads were tested for transpeptidation by the on-bead reactions (Abu, l-Aminobutyric acid; Aoa, l-Aminooxyacetic acid; Cit, l-Citrulline; Dab, l-Diaminobutyric acid; Dap, l-Diaminopropionic acid; hLys, l-Homolysine; Nle, l-Norleucine; Orn, l-Ornithine; Lys, l-Lysine). The error bars show averages ±1 SD (n = 3). (**B**) Various unnatural amino acids mimicking phenylalanine at the X_2_ position of the KX_2_ beads were tested for transpeptidation by the on-bead reactions (1Nal, 1-Naphthyl-l-alanine; 2Nal, 2-Naphthyl-l-alanine; 3Br, 3-Bromo-l-phenylalanine; 4Br, 4-Bromo-l-phenylalanine; B-hPhe, l-beta-homophenylalanine; Bip, 4-l-Biphenylalanine; Bpa, 4-l-Benzoyl-phenylalanine; Bth, 3-l-Benzothiazolyl-alanine; d-hPhe, d-homophenylalanine; Cha, 3-Cyclohexyl-l-alanine; Pal, 4-Pyridyl-l-alanine; hPhe, l-homophenylalanine; Phg, l-Phenylglycine; Tic, 1,2,3,4-Tetrahydroisoquinoline-3-carboxylic acid; mTyr, O-methyl-l-tyrosine; Phe, l-Phenylalanine). The error bars show averages ±1 SD (n = 3). (**C**) Four combinations of l- and d-amino acids in the KF dipeptide were tested for transpeptidation (10 µM DegP and 100 µM 18–58) in the context of KFK(biotin) (10 mM), and quantitatively analyzed by HPLC (n = 3). (**D**) Combinations of Dap, Lys, and Orn at the X_1_ position with Phe and hPhe at the X_2_ position were tested for transpeptidation (10 µM DegP and 100 µM 18–58) in the context of X_1_X_2_K(biotin) (20 mM), and they were quantitatively analyzed by HPLC (n = 3). (**E**,**G**) Transpeptidation reactions (10 µM DegP and 100 µM 18–58) were analyzed by HPLC with various concentrations (2.5–20 mM) of KFK(biotin) and OFK(biotin) ((**E**); n = 1) or with 10 mM of KF**X**K(biotin), in which **X** represents various amino acids ((**G**); n = 1). (**F**) The relative fluorescence of the p23 (Abz-GNWVSAA*KFEY^NO2^SKNTQDYGILQI, where * is the cleavage site) cleavage reactions (1 µM DegP and 40 µM p23) was monitored in the presence of increasing amounts of the quencher probe (KFY^NO2^). The error bars show averages ± 1 SD (n = 3). Abz, 2-amino benzoic acid; Y^NO2^, 3-nitrotyrosine. (**H**) KFK(biotin) (20 mM) was added before or after the cleavage reactions (5 µM DegP and 50 µM 18–58 in 20 µL solution), and the reaction products were analyzed by MALDI-TOF-MS. Representative spectra are shown (n = 3).

To investigate whether unnatural amino acids can increase the efficiency of transpeptidation, we prepared bead-attached peptides carrying various unnatural amino acids, many of which mimicked lysine or phenylalanine at the X_1_ or X_2_ position, respectively (Figure S4). On-bead reactions revealed that lysine (Lys) is the most preferred amino acid at the X_1_ position, followed by l-ornithine (Orn) and 2,3-diaminopropionic acid (Dap) (Figure 5A). Those with phenylalanine mimics at the X_2_ position showed that phenylalanine is the most favored, followed by l-homophenylalanine (hPhe) and 4-benzoyl-l-phenylalanine (Bpa) (Figure 5B or Figure S5).

To quantitatively analyze the transpeptidation reaction, we prepared peptides in the X_1_X_2_K(biotin) context and measured the percentage of addition by HPLC. The combination of l- and d-amino acids in the KF dipeptide at the X_1_X_2_ site did not show any significant differences in addition efficiency among the four combinations (Figure 5C). As expected, the addition product of ll-peptide, but not the one of dd-peptide, was cleaved after lysine at the X_1_ position by trypsin, supporting the presence of d-lysine (Figure S6). Therefore, we focused on peptides containing only l-amino acids. The combination of some unnatural amino acids showed that those with Lys_Phe (KF) and Orn_Phe (OF) have the best efficiency (Figure 5D or Figure S4). Indeed, titration with various concentrations of KFK(biotin) and OFK(biotin) showed almost same amount of addition products (Figure 5E). The fluorescence resonance energy transfer (FRET) experiment with a substrate carrying the N-terminal fluorophore (2-aminobenzoic acid, Abz) and a nucleophile bearing the C-terminal quencher (3-nitrotyrosine, Y^NO2^) showed that higher concentrations of the quencher nucleophile significantly reduced the fluorescence intensity, further confirming the addition of the nucleophile (Figure 5F). To see if longer peptides work better, we tested KF**X**K(biotin) in which one additional amino acid was inserted and found that the addition efficiency was not better than KFK(biotin) (Figure 5G). Finally, when we added KFK(biotin) either before or after the cleavage reaction, the latter did not show the addition product, demonstrating that the addition reaction is not a result of reverse proteolysis in which the hydrolyzed peptides are ligated via condensation (Figure 5H). Collectively, KFK(biotin) and OFK(biotin) are the best nucleophilic probe with an affinity tag among those we tested.

### 2.4. Selective Enrichment of the Cleavage Products with Model Substrates

**Figure 6:**
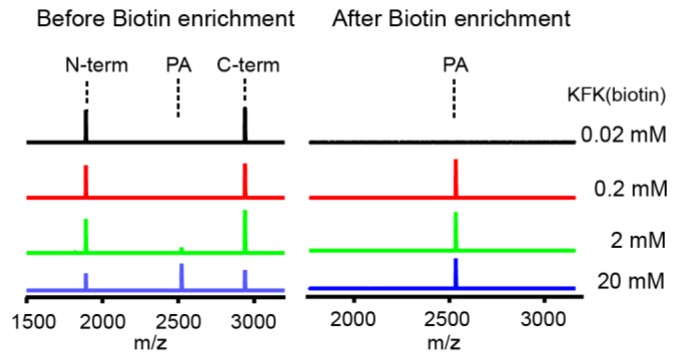
The cleavage reactions (10 µM DegP and 100 µM 18–58) with various concentrations of KFK(biotin) (0.02–20 mM) were analyzed by MALDI-TOF-MS before (left panel) and after (right panel) biotin enrichment via streptavidin-linked magnetic beads. Representative spectra are shown (n = 3).

The probe we found above, KFK(biotin), has a biotin that is covalently linked to the ε-amine group of the C-terminal lysine. To test if this biotin allows the affinity purification of the probe-addition product, we performed reactions with various concentrations of the KFK(biotin) probe (0.02–20 mM). We were able to detect the peak for the addition product in MALDI spectra with a probe of a minimum of 2 mM (Figure 6, left panel). However, the purification with streptavidin-linked magnetic beads led to selective enrichment of the addition product, and in particular, the experiment with even 0.2 mM of probe, with which the addition efficiency was about one percent, clearly showed the addition product (Figure 6, right panel). This result demonstrates that it is possible to selectively enrich the substrate probe fusion peptides with an affinity tag.

**Figure 7:**
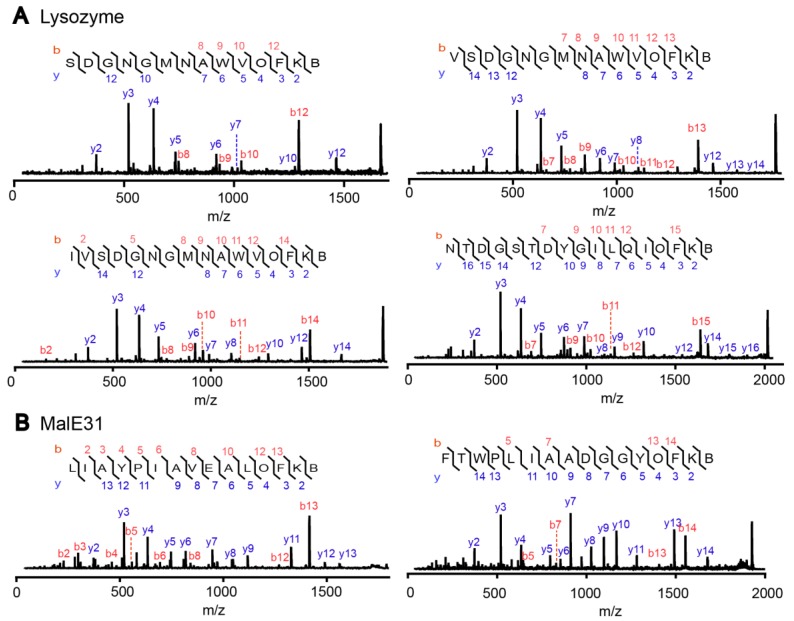
The probe addition products of the model protein substrates lysozyme (**A**) and MalE31 (**B**) were identified by MALDI-TOF-MS/MS. Misfolded lysozyme and MalE31 (80 µM each) were degraded by DegP (10 µM) in the presence of the OFK(biotin) probe (10 mM). The reaction products were further cleaved by trypsin and subjected to MALDI-TOF-MS in which several peaks were further analyzed by MS/MS.

DegP can degrade various misfolded proteins, of which misfolded lysozyme and a defective folding mutant of MalE, MalE31, are known to be good substrates [19,23]. We degraded these proteins with DegP by adding dithiothreitol (DTT; 5 mM) to the lysozyme-containing reaction solution or by diluting the urea-dissolved MalE31 into the reaction solution in the presence of the OFK(biotin) probe (10 mM; Figure S7). DTT induces misfolding of lysozyme by reducing disulfide bonds. The reaction solutions were treated with trypsin and subjected to biotin enrichment, followed by MALDI-TOF-MS/MS analysis. We found four and two probe addition products from lysozyme and MalE31 degradation, respectively, as shown in the MS/MS spectra, in which distinct fragmentation patterns for the C-terminal addition of the OFK(biotin) probe were observed (Figure 7). This result demonstrates that the probe is able to trap the cleaved peptides from protein substrates as well as the model peptide, 18–58.

## 3. Discussion

Proteases have evolved to mediate the hydrolysis of peptide bonds, which is an energetically downhill process. Therefore, transpeptidation by proteases normally occurs as a rare event or at a low efficiency. Here, we found nucleophilic probes that can readily react with acyl-enzyme intermediates and efficiently trap the cleavage products of a model protease, DegP, in a normal buffer condition. The OBOC-based on-bead screening approach was successfully applied to identify KFK(biotin) and OFK(biotin) as good probes which are covalently linked to the C-termini of the cleaved peptides and allow selective enrichment of the ligated peptides. This probe should react with the acyl-enzyme intermediates for transpeptidation before water molecules do for hydrolysis at a very high concentration (~55.5 M in aqueous solutions), but only 20 mM of the probe is required to obtain 20–25% of the addition product of the model substrate, indicating that this probe can compete with a water molecule at a 1000-fold lower concentration and generate a comparable amount of the transpeptidation product over the hydrolysis product. Also, we showed that the enrichment of the addition product allows its detection at a much lower probe concentration. We suggest that many other proteases acting via acyl-enzyme intermediates can also perform transpeptidation in the presence of a proper nucleophilic molecule and that these probes will help in the characterization of the substrates of proteases.

This study differs from the previous examples of protease-mediated transpeptidation or peptide ligation in the identification of efficient nucleophilic probes. Those with trypsin, V8 protease, Glu-C, bleomycin hydrolase, and human proteasome showed peptide ligation between cleaved peptides at a low efficiency [4,5,6,7,8,12,13]. Subtiligase, a mutant variant of subtilisin BPN’, normally uses activated ester substrates for the selective labeling of proteins with free N-terminal α-amine, but our probe was applied to a wild-type protease for the selective C-terminal labeling of cleaved peptides [9,10,11]. Many other examples of peptide ligation using proteases require activated ester substrates and harsh conditions, such as organic solvents, high pH, and high reactant concentrations, and are mainly applied to peptide synthesis in vitro [24,25]. Our study mainly concerned the selective ligation of the probe to the cleaved peptides under normal conditions, and therefore, this approach may have applications both in vitro and in vivo for substrate characterization.

It is unknown how exactly the probe triggers transpeptidation over hydrolysis, but it is likely that the probe transiently binds to the site near the active site and is positioned to readily attack acyl-enzyme intermediates [26,27]. In particular, two amino acids in the probe may function as the P1′ and P2′ residues which fit the S’-positions of the enzyme that are originally occupied by the C-terminal fragment of the substrate. This idea is consistent with our result whereby a specific residue, lysine for the DegP probe, is preferred at the N-terminal position of the probe for efficient transpeptidation. Therefore, the P1′ specificity of the protease may be a good starting point to find a good probe.

It has been suggested that peptide splicing in the human proteasome may generate a unique set of antigens with distinct immunological properties and may facilitate the development of vaccines and cancer immunotherapies [15]. The potential role of transpeptidation by other proteases is unknown, but one possibility may be to help proteolysis by removing relatively long-lived acyl-enzyme intermediates that may slow down the reaction. This idea suggests that the probe may be added more efficiently to a specific set of peptides with slow cleavage kinetics. In this case, the transpeptidiation efficiency may be dependent on the sequence of substrates as well as the probe. Further studies about the kinetics of hydrolysis and transpeptidation with various substrates will be required to support this idea.

## 4. Materials and Methods

### 4.1. Materials and Equipment

General chemicals and reagents were purchased from Fisher Acros (Hampton, NH, USA). Isothiocyanobenzyl-NTA was purchased from Biomass (Rockville, MD, USA). Tentagel S NH_2_ resin was purchased from RAPP polymer (Tuebingen, Germany). Dynabeads^TM^ MyOne^TM^ Streptavidin C1, Streptavidin-alkaline phosphatase (SAAP), and 5-bromo-4-chloro-3-indolyl-phosphate (BCIP) were obtained from Thermofisher (Hampton, NH, USA). Amino acids, coupling reagents, and biotin were purchased from GL-Biochem. BSA (Shanghai, China), lysozyme, and dipeptides were obtained from Sigma-Aldrich (St. Louis, MO, USA). Ni Sepharose 6 FastFlow beads were obtained from GE Healthcare (Chicago, IL, USA).

MALDI-TOF-MS and MALDI-TOF-MS/MS analysis were performed by a Bruker Micro-flex MALDI-TOF mass spectrometer (Billerica, MA, USA) and a Bruker Ultra-flextreme MALDI-TOF/TOF mass spectrometer. The HPLC analysis and HPLC purification were performed using Agilent 1260 Infinity (Agilent, Santa Clara, CA, USA). Positive bead (blue beads) screening was performed with a Nikon SMZ 745T microscope (Nikon, Tokyo, Japan).

### 4.2. Expression and Purification of Proteins and a Peptide

DegP^WT^ and the model substrate (18–58) were prepared as previously described [19,28]. The plasmid expressing 6His-MalE31 was constructed by mutating G32/I33 to D32/P33 in the MalE-expressing plasmid. Cells harboring the 6His-MalE31 plasmid were grown at 30 °C in 1 L LB medium with kanamycin (50 μg/mL). The 6His-MalE31 expression was induced at A_600_ ~0.5 with isopropyl-1-thio-β-d-galactopyranoside (IPTG, 0.1 mM) for 4–5 h. Cells were harvested, resuspended in 30 mL of 25 mM Tris buffer (pH 7.5), lysed by sonication, and centrifuged at 13,000 rpm for 30 min. The pellets were resuspended in 10 mL of the above buffer containing 2% Triton X-100. After centrifugation, inclusion bodies (pellets) were solubilized by urea Ni-column wash buffer (8 M urea, 300 mM NaCl, Tris 20 mM, pH 6.0). 6His-MalE31 was bound to Ni Sepharose 6 FastFlow beads, washed with wash buffer, and eluted with buffer B (8 M Urea, 300 mM NaCl, 20 mM Tris, pH 4.0). The eluted solution was concentrated with Amicon centrifugal filter (Millipore, Burlington, MA, USA).

### 4.3. Peptide Synthesis

OBOC peptide libraries were constructed using solid phase peptide synthesis. Tentagel S NH_2_ resin (0.7 g) was washed and soaked with 10 mL of 1:1 DMF/DCM for 20 min in a reaction vessel. Fmoc-Met-OH (5 equiv.) and HATU/DIEA (5/10 equiv.) dissolved in DMF were added to the reaction vessel and the reaction solution was incubated at room temperature for 30 min. Beads were washed three times with DMF and DCM. After the completion of coupling, monitored by ninhydrin and TNBS tests, beads were treated with capping solution (DMF/acetic anhydride/DIEA in a 9:1:0.05 ratio) for 8 min to block the unreacted amines. Beads were mixed with 20% (*v*/*v*) piperidine in DMF for 20 min to remove the Fmoc group and washed three times with DMF and DCM. The above coupling, capping, and deprotection steps were repeated to add four more amino acids, resulting in the AAARM peptide. Beads were washed six times with DMF and DCM, split into 19 equal portions in new reaction vessels. Beads were individually coupled to 19 amino acids (except cysteine) and subjected to the Fmoc deprotection and the DMF/DCM wash. All the beads were combined in one reaction vessel, split again into 19 equal portions, and subjected to the second coupling reactions with 19 amino acids. The N-terminal Fmoc group was removed with 20% (*v*/*v*) piperidine in DMF, and the side chain protection groups of the peptide library were removed with cleavage cocktail (95% TFA, 2.5% deionized water, 2.5% triisopropyl silane) for 4 h at room temperature. Finally, beads were washed with DMF and DCM and became ready for the on-bead screening experiment.

The X_1_X_2_K(biotin) peptides were synthesized using solid phase peptide synthesis. Fmoc-Lys(mtt)-OH (5 equiv.) and DMAP/DIC (0.1 and 4 equiv., respectively) were added to the washed and soaked Wang resin and mixed in DMF at room temperature for 30 h. After capping and washing beads, the 4-methyltrityl (MTT) protecting group was cleaved using 2% TFA in DCM for 30 min. The beads were washed with DMF and DCM, and mixed with the activated biotin solution, which was prepared by mixing 5 equiv. (d)-Biotin, 4 equiv. HATU, and 10 equiv. DIEA in 10 mL DMF/DMSO (1:1). After the beads were washed, two more rounds of the deprotection and amino acid coupling were performed. After the last coupling, a cleavage cocktail was applied to the resin for 2 h. The solution was then evaporated using nitrogen stream over the tube. For each 1 mL of the solution, 10 mL of ice-cold ether/hexane (1:1) was added for peptide precipitation. The precipitated peptides were centrifuged and washed with cold precipitation solution to remove the scavenger. Peptides were dissolved in DMSO, diluted with 0.05% TFA in water, and purified by HPLC and lyophilization.

The N-terminally biotinylated substrate of DegP (Biotin-SLGNWVSAAKFESNFNTQDYGILQI) and the p23 peptide (Abz-GNWVSAA KFEY^NO2^SKNTQDYGILQI; Abz, 2-aminobenzoic acid; Y^NO2^, 3-nitrotyrosine) were synthesized using solid phase peptide synthesis, as described above.

### 4.4. On-Bead Reaction and Screening

Two hundred and fifty nanomoles of Tentagel beads (64,790 beads) in 75 µL of NTA binding buffer (50 mM phosphate, 300 mM NaCl, pH 8.5) was mixed with 25 µL of Isothiocyanobenzyl-NTA (ITC-Bz-NTA; 100 μM) in DMSO and incubated at 37 °C for 1 h with shaking. Beads were washed five times with NTA binding buffer and mixed with 5 equivalent NiSO_4_ at room temperature for 30 min with shaking. Beads were washed with enzyme binding buffer (50 mM phosphate, 300 mM NaCl, 20 mM imidazole, pH 8.0) and mixed with 0.2 nM 6His-DegP at room temperature for 30 min in 100 μL of enzyme binding buffer. After washing with enzyme binding buffer, beads were incubated with 5 nM biotinylated peptide containing blocking buffer (50 mM phosphate, 100 mM NaCl, 4% BSA, pH 8.0) at 37 °C for 2 h with shaking. The reaction solution was decanted, and the beads were washed with the blocking buffer and SAAP buffer (30 mM Tris, 1 M NaCl, pH 7.4). Small amounts of beads (~200 beads each group) were transferred to a transparent 96-well plate with another 20 μL of SAAP buffer, and mixed with 1 μL of SAAP solution (1 mg/mL) for 10 min at 4 °C. After the solution was removed, the beads were washed with the SAAP buffer and BCIP staining buffer (30 mM Tris, 100 mM NaCl, 5 mM MgCl_2_, 2 μM ZnCl_2_, pH 8.4). One hundred microliters of BCIP staining buffer and 20 μL of 5 mg/mL BCIP were added to the beads, and the plate was incubated for about 30 min on a shaker. Some beads turned blue. The staining reaction was quenched by adding 100 µL of 1 M HCl. Beads were transferred to a transparent film for counting or picking up the blue beads using a microscope. For peptide cleavage, each blue bead was transferred into a microtube, washed with water, and treated with 20 μL of the CNBr solution (40 mg/mL in 70% (*v*/*v*) TFA and H_2_O) overnight. The solvent was evaporated with nitrogen stream, and 10 μL of the solution (10% Acetonitrile, 0.05% TFA) was added into each tube to dissolve the peptides. After desalting with C18 Ziptip, peptides were analyzed by MALDI-TOF-MS.

### 4.5. Reactions in Solution and HPLC Analysis

The small-scale reaction mixture (10–20 µL) contained DegP (2 μM), a peptide substrate (18–58; 20 μM), and various nucleophiles at the indicated concentrations in reaction buffer (50 mM phosphate, 100 mM NaCl, pH 8.0). The reaction tube was incubated at room temperature for 2 h. TFA was added at a final concentration of 0.1% (*v*/*v*) to quench the reaction. After desalting with C18 Ziptip, peptides were analyzed by MALDI-TOF-MS.

For the HPLC analysis, the peptide substrate (18–58; 100 µM) was added to the reaction solution (100 µL final) containing DegP (10 µM) and nucleophiles (indicated concentration) in the reaction buffer (50 mM phosphate, 100 mM NaCl, pH 8.0). After incubation at room temperature for 2 h, the reaction was quenched by adding TFA at a final concentration of 0.1% (*v*/*v*). The reaction solution was desalted with C18 Ziptip, diluted 10-fold with HPLC solvent A (0.05% TFA in water), and analyzed by HPLC. A C18 reverse phase HPLC column (ZORBAX SB-C18 4.6 × 250 mm, Santa Clara, CA, USA) was used to separate the peptides using a 10–40% acetonitrile gradient for 30 min at a flow rate of 1 mL/min. Each peak was analyzed by MALDI-TOF-MS. The probe addition efficiency was quantified by measuring the peak areas of the N-terminal fragment and the addition product.

### 4.6. FRET Assay

Reaction solutions (10 µL) containing DegP (1 μM), KFY or KFY^NO2^ (0–40 mM), and p23 (40 μM) in reaction buffer (50 mM phosphate, 100 mM NaCl, pH 8.0) were incubated at room temperature for 2 h. The probe addition was monitored after the reaction by measuring fluorescence (excitation at 320 nm; emission at 430 nm) using an Infinite F200Pro microplate reader (Tecan, Männedorf, Swiss).

### 4.7. Enrichment of the Addition Products

For enrichment of the addition product from the model peptide, the reaction solutions (100 µL) containing 18–58 (100 µM), DegP (10 µM), and OFK(biotin) (0.02, 0.2, 2, or 20 mM) were prepared in reaction buffer (50 mM phosphate, 100 mM NaCl, pH 8.0). After incubation at room temperature for 2 h, the solution was passed through a C18 Ziptip for peptide binding. The Ziptip was washed eight times with 200 μL of 10% acetonitrile/0.05% TFA (*v*/*v*) solution to remove the excessive probe, and peptides were eluted with 10 μL of 80% acetonitrile/0.05% TFA (*v*/*v*). The eluted solution was dried, and peptides were dissolved in binding buffer (50 mM phosphate, 300 mM NaCl, pH 7.4). Twenty microliters of streptavidin Dynabead C1 (Thermofisher) was washed three times with binding buffer and added to the peptide solution. After incubation at room temperature for one hour with shaking, the beads were washed five times with binding buffer. Biotinylated peptides were eluted by boiling at 95 °C for 10 min with 20 μL of elution solution (95% Formamide, 10 mM EDTA, pH 8.2). The eluted solution was transferred to a microtube, diluted with solvent A (200 μL H_2_O, 0.05% TFA (*v*/*v*)), and desalted with C18 Ziptip. Peptides were analyzed by MALDI-TOF-MS.

For the enrichment of the addition product from the model proteins, lysozyme (80 µM) and MalE31 (80 µM final, diluted from 2 mM solution in 8 M urea) were individually degraded by DegP (10 µM) in the presence of OFK(biotin) (10 mM) and DTT (5 mM) in reaction buffer (50 mM phosphate, 100 mM NaCl, pH 8.0; 100 µL final volume). The reaction solutions were incubated at 42 °C (lysozyme) or at room temperature (MalE31) for 4 h. Protein degradation was confirmed by SDS-PAGE. The reaction solutions were diluted 20-fold with the reaction buffer (50 mM phosphate, 100 mM NaCl, pH 8.0), treated with trypsin in a final protein:protease ratio of 20:1 (*w*/*w*), and incubated at room temperature overnight. The trypsin digestion was stopped by adding 0.1% TFA. The solutions were loaded onto a Ziptip streptavidin Dynabead C1, and then to a second Ziptip for the enrichment of biotinylated peptides, as described above. Peptides were analyzed by MALDI-TOF-MS/MS (Bruker Daltonics) in which the tandem mass spectra were used to identify the probe-addition products.

## Supplementary Materials

The following are available online. Figure S1: MALDI spectra of the control experiments in which nucleophile, enzyme, or both nucleophile and enzyme were absent, Figure S2: Microscopic images of beads after on-bead reaction, Figure S3: MALDI spectra of peptides that were cleaved from blue beads, Figure S4: Chemical structures of probes used in Figure 5, Figure S5: The full time-course data for Figure 5B, Figure S6: MALDI spectra of the trypsin-treated addition products, and Figure S7: SDS-PAGE of the degradation of two misfolded proteins.

## References

[1] Drag M., Salvesen G.S. (2010). Emerging principles in protease-based drug discovery. Nat. Rev. Drug Discov..

[2] Deu E., Verdoes M., Bogyo M. (2012). New approaches for dissecting protease functions to improve probe development and drug discovery. Nat. Struct. Mol. Biol..

[3] Rawlings N.D., Waller M., Barrett A.J., Bateman A. (2014). MEROPS: The database of proteolytic enzymes, their substrates and inhibitors. Nucleic Acids Res..

[4] Canova-Davis E., Kessler T.J., Ling V.T. (1991). Transpeptidation during the analytical proteolysis of proteins. Anal. Biochem..

[5] Lippincott J., Hess E., Apostol I. (1997). Mapping of recombinant hemoglobin using immobilized trypsin cartridges. Anal. Biochem..

[6] Schaefer H., Chamrad D.C., Marcus K., Reidegeld K.A., Bluggel M., Meyer H.E. (2005). Tryptic transpeptidation products observed in proteome analysis by liquid chromatography-tandem mass spectrometry. Proteomics.

[7] Fodor S., Zhang Z. (2006). Rearrangement of terminal amino acid residues in peptides by protease-catalyzed intramolecular transpeptidation. Anal. Biochem..

[8] Zheng W., Johnston S.A., Joshua-Tor L. (1998). The unusual active site of Gal6/bleomycin hydrolase can act as a carboxypeptidase, aminopeptidase, and peptide ligase. Cell.

[9] Abrahmsen L., Tom J., Burnier J., Butcher K.A., Kossiakoff A., Wells J.A. (1991). Engineering subtilisin and its substrates for efficient ligation of peptide bonds in aqueous solution. Biochemistry.

[10] Chang T.K., Jackson D.Y., Burnier J.P., Wells J.A. (1994). Subtiligase: A tool for semisynthesis of proteins. Proc. Natl. Acad. Sci. USA.

[11] Mahrus S., Trinidad J.C., Barkan D.T., Sali A., Burlingame A.L., Wells J.A. (2008). Global sequencing of proteolytic cleavage sites in apoptosis by specific labeling of protein N termini. Cell.

[12] Vigneron N., Stroobant V., Chapiro J., Ooms A., Degiovanni G., Morel S., van der Bruggen P., Boon T., van den Eynde B.J. (2004). An antigenic peptide produced by peptide splicing in the proteasome. Science.

[13] Hanada K.-I., Yewdell J.W., Yang J.C. (2004). Immune recognition of a human renal cancer antigen through post-translational protein splicing. Nature.

[14] Warren E.H., Vigneron N.J., Gavin M.A., Coulie P.G., Stroobant V., Dalet A., Tykodi S.S., Xuereb S.M., Mito J.K., Riddell S.R. (2006). An antigen produced by splicing of noncontiguous peptides in the reverse order. Science.

[15] Liepe J., Marino F., Sidney J., Jeko A., Bunting D.E., Sette A., Kloetzel P.M., Stumpf M.P., Heck A.J., Mishto M. (2016). A large fraction of HLA class I ligands are proteasome-generated spliced peptides. Science.

[16] Clausen T., Kaiser M., Huber R., Ehrmann M. (2011). HTRA proteases: Regulated proteolysis in protein quality control. Nat. Rev. Mol. Cell Biol..

[17] Krojer T., Sawa J., Schafer E., Saibil H.R., Ehrmann M., Clausen T. (2008). Structural basis for the regulated protease and chaperone function of DegP. Nature.

[18] Krojer T., Pangerl K., Kurt J., Sawa J., Stingl C., Mechtler K., Huber R., Ehrmann M., Clausen T. (2008). Interplay of PDZ and protease domain of DegP ensures efficient elimination of misfolded proteins. Proc. Natl. Acad. Sci. USA.

[19] Kim S., Grant R.A., Sauer R.T. (2011). Covalent linkage of distinct substrate degrons controls assembly and disassembly of DegP proteolytic cages. Cell.

[20] Lam K.S., Lebl M., Krchňák V. (1997). The “one-bead-one-compound” combinatorial library method. Chem. Rev..

[21] Lehman A., Gholami S., Hahn M., Lam K.S. (2006). Image subtraction approach to screening one-bead-one-compound combinatorial libraries with complex protein mixtures. J. Comb. Chem..

[22] Kunys A.R., Lian W., Pei D. (2012). Specificity Profiling of Protein-Binding Domains Using One-Bead-One-Compound Peptide Libraries. Curr. Protoc. Chem. Biol..

[23] Betton J.-M., Sassoon N., Hofnung M., Laurent M. (1998). Degradation versus aggregation of misfolded maltose-binding protein in the periplasm of Escherichia coli. J. Biol. Chem..

[24] Jakubke H.D., Kuhl P., Könnecke A. (1985). Basic principles of protease-catalyzed peptide bond formation. Angew. Chim. Int. Ed. Engl..

[25] Schellenberger V., Jakubke H.D. (1991). Protease-catalyzed kinetically controlled peptide synthesis. Angew. Chim. Int. Ed. Engl..

[26] Riechmann L., Kasche V. (1985). Peptide synthesis catalyzed by the serine proteinases chymotrypsin and trypsin. Biochim. Biophys. Acta BBA.

[27] Berkers C.R., de Jong A., Ovaa H., Rodenko B. (2009). Transpeptidation and reverse proteolysis and their consequences for immunity. Int. J. Biochem. Cell Biol..

[28] Kim S., Sauer R.T. (2012). Cage assembly of DegP protease is not required for substrate-dependent regulation of proteolytic activity or high-temperature cell survival. Proc. Natl. Acad. Sci. USA.

