# Supplementary material for: Identification of Nucleophilic Probes for Protease-Mediated Transpeptidation
Source: Molecules. 2018 Aug 22;23(9):2109. doi: 10.3390/molecules23092109 (PMC6225416; doi:10.3390/molecules23092109)
Supplement: Supplementary file 1 [file molecules-23-02109-s001.pdf]

Supplementary Material for

Identification of nucleophilic probes for protease-mediated transpeptidation

Ga-eul Eom and Seokhee Kim*

Department of Chemistry, Seoul National University,

1 Gwanak-ro, Gwanak-gu, Seoul 08826, South Korea

* Corresponding author: seokheekim@snu.ac.kr; +82-2-880-4123 (phone); +82-2-875-6636 (fax)

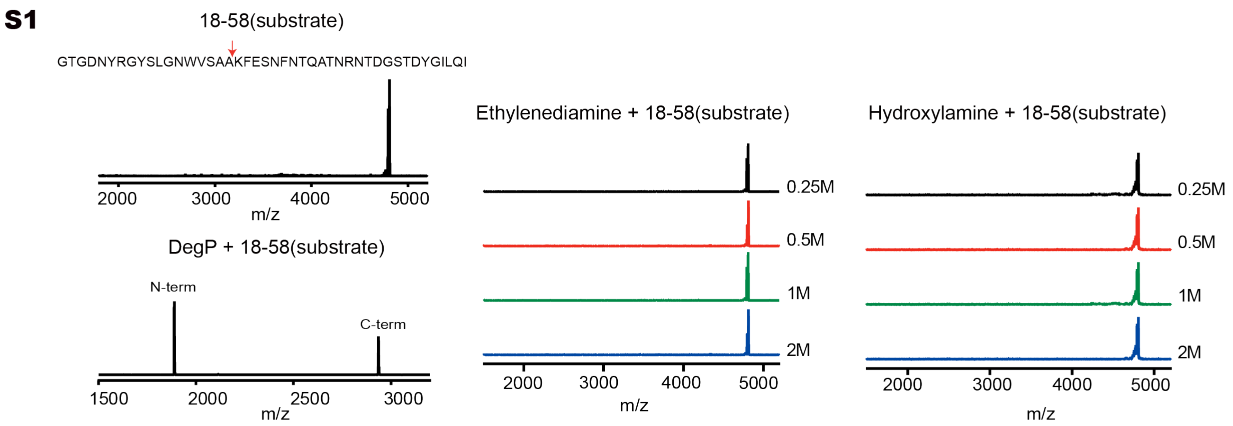

Figure S1. MALDI spectra of the control experiments in which nucleophile, enzyme, or both nucleophile and enzyme were absent. The reaction conditions were same as that of Figure 2A.

S2

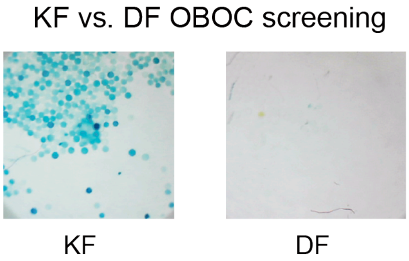

Figure S2. Microscopic images of beads after the on-bead reactions. They revealed that more KF-beads were blue than DF-beads in the on-bead reaction.

S3 Bead CNBr cleavage(KXAAAR-M)

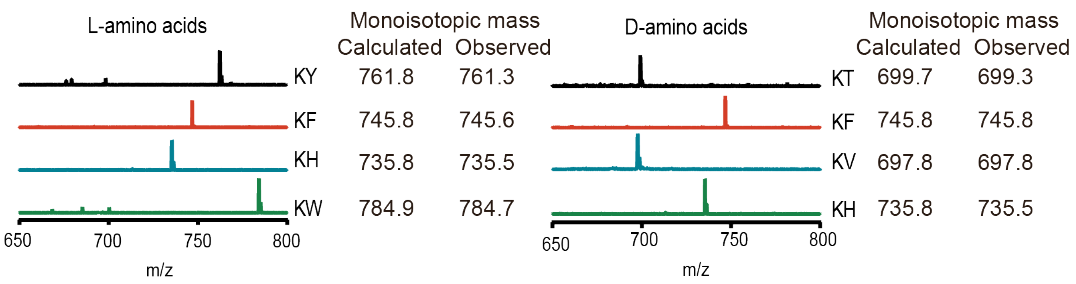

Figure S3. MALDI spectra of peptides that were cleaved from blue beads. Calculated and observed masses from the MALDI spectra are shown.

S4

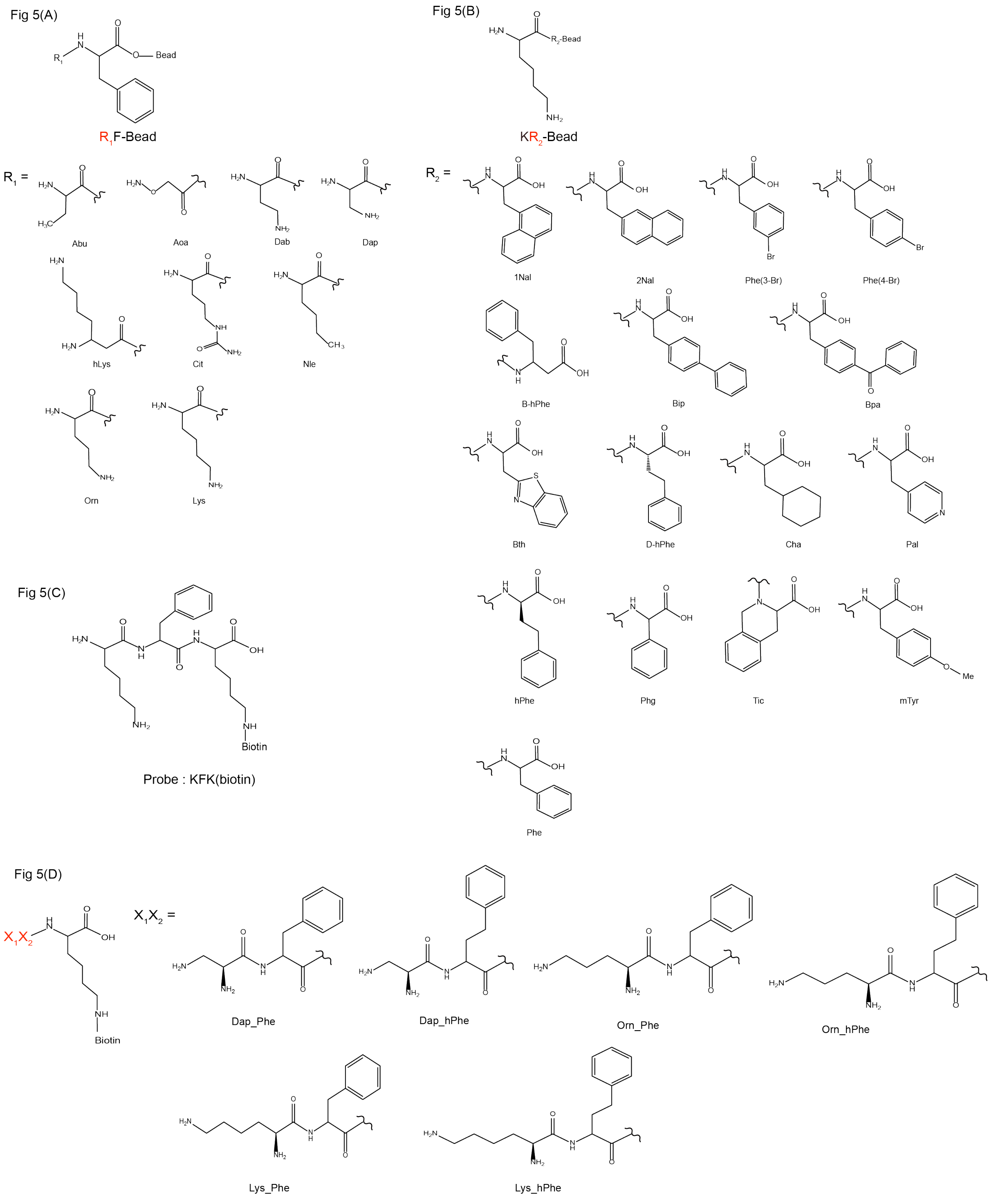

Figure S4. Chemical structures of probes used in Figure 5.

S5

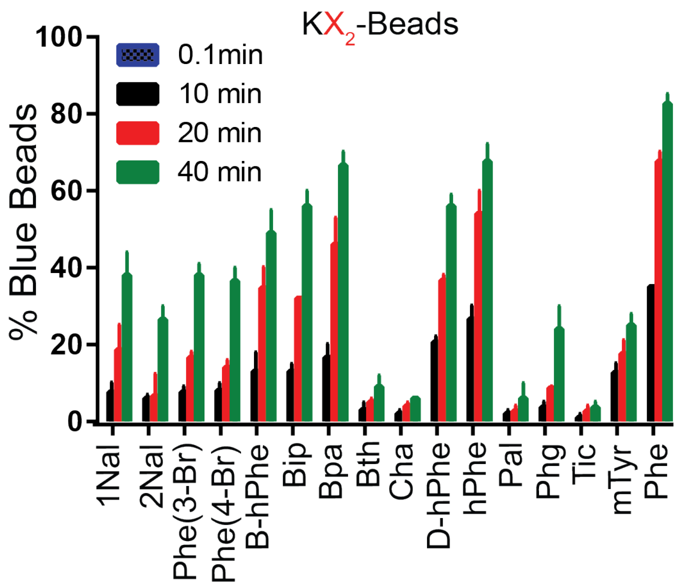

Figure S5. The full time-course data for Figure 5B.

S6

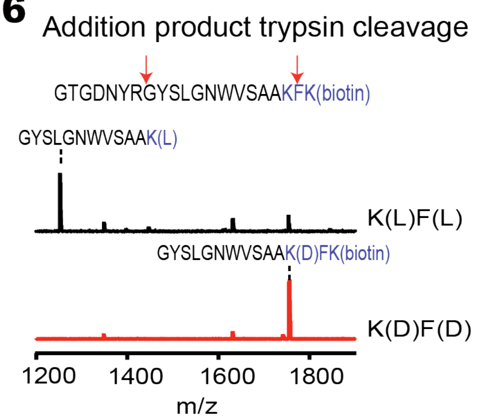

Figure S6. MALDI spectra of the trypsin-treated addition products. Two addition products with L- or D-form of KFK(biotin), K(L)F(L)K(biotin) or K(D)F(D)K(biotin), were cleaved by trypsin and analyzed by MALDI. The scissile bond after K(L) and F(L), not the one after K(D) and F(D), was efficiently cleaved by trypsin.

S7

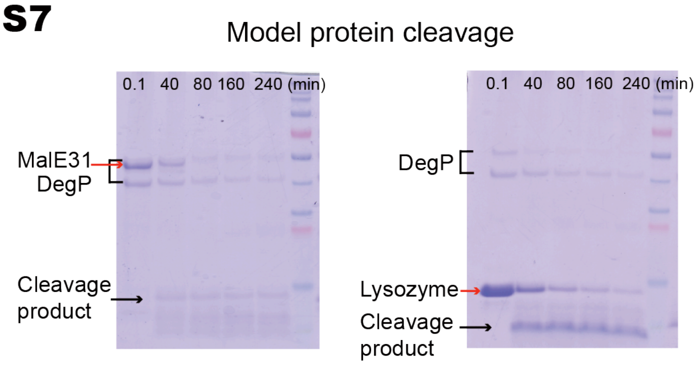

Figure S7. SDS-PAGE of the degradation of two misfolded proteins. Misfolded MalE31 (left) and lysozyme (right) were degraded by the DegP protease, as shown in SDS-PAGE.
